# Measuring the effects of guided clinical reasoning on the Advanced Nursing Process quality, on nurses’ knowledge and attitude: Study protocol

**DOI:** 10.1002/nop2.299

**Published:** 2019-06-02

**Authors:** Claudia Leoni‐Scheiber, Hanna Mayer, Maria Müller‐Staub

**Affiliations:** ^1^ Institute of Nursing Science University Vienna Vienna Austria; ^2^ Lectoraat Nursing Diagnostics Hanze University Groningen Groningen the Netherlands; ^3^ City Hospital Waid Zurich Switzerland

**Keywords:** Advanced Nursing Process, Experimental studies, Guided clinical reasoning, Instrument Quality of Diagnoses, Interventions, and Outcomes (Q‐DIO), Nurse attitudes, Nursing knowledge, Nursing records, Positions on Nursing Diagnoses Scale, Record review, Standardized nursing language

## Abstract

**Aim:**

This article is a report of a study protocol designed to examine the effects of guided clinical reasoning on the quality of the Advanced Nursing Process—the evidence‐based version of the traditional nursing process. It aims to describe the theoretical framework—Kirkpatrick's evaluation model, the key concepts and the instruments for the planned study.

**Design:**

A complex experimental intervention study using data and method triangulation is proposed.

**Methods:**

Registered Nurses (*N* = 92), nursing records (*N* = 180) and 24 patients will be included. Nurses’ knowledge and attitude will be evaluated by questionnaires/tests, their clinical performance by observations. Patients’ perspective will be addressed by qualitative interviews and patient records by using the instrument Quality of Diagnoses, Interventions and Outcomes revised (Q‐DIO R).

**Discussion:**

Kirkpatrick's model (including quantitative and qualitative methods) is providing evaluations from different perspectives on the quality of the Advanced Nursing Process and on intervention effects.

## 1 INTRODUCTION

Nursing care must be transparently described due to financial, legal (Health Insurance Act—KVG, 2016) and professional requirements (Müller‐Staub, Abt, Brenner, & Hofer, 2015). This can be achieved by applying and documenting the nursing process with standardized nursing languages (SNLs) (Escalada‐Hernández et al., 2015; Estrada & Dunn, 2012; Hariyati, Yani, Eryando, Hasibuan, & Milanti, 2016; Jones, Lunney, Keenan, & Moorhead, 2010; Pérez Rivas et al., 2016). This approach increases patient safety and leads to better nursing‐sensitive outcomes (Pérez Rivas et al., 2016). However, nurses face challenges in clearly documenting the nursing process. Several studies on nursing process documentation have shown clinically relevant deficits, with the consequences of impaired patient well‐being, lower care quality, including risks to patient safety (De Marinis et al., 2010; Gershater, Pilhammar, & Alm Roijer, 2010; Pereira et al., 2015; Zegers et al., 2011). The application of the nursing process based on SNLs is a challenging task and should be fostered through educational interventions. guided clinical reasoning (GCR) could be a useful method for this purpose (Müller‐Staub, Needham, Odenbreit, Lavin, & Achterberg, 2008). The aim of this protocol is to describe a comprehensive methodological paper demonstrating a research plan including its theoretical framework—Kirkpatrick's evaluation model—the key concepts Advanced Nursing Process and the study intervention GCR along with the instruments for data collection.

## 2 BACKGROUND

The nursing process provides a systematic approach for nursing care (American Nurses Association [ANA], 2015a). This relationship and problem‐solving process were further developed into an extended, deepened and research‐based version, so‐called Advanced Nursing Process. It “consists of defined, validated concepts. It includes assessment, nursing diagnoses, nursing interventions and nursing outcomes that are rooted in scientifically based nursing classifications” (Müller‐Staub et al., 2015, p. 13). Compared with the “traditional nursing process” without SNLs, the Advanced Nursing Process contains valid assessment tools and evidence‐based diagnoses, intervention and outcome concepts (=SNLs) as described in nursing classifications. The classification systems that best meet literature‐based validity and reliability criteria are the NANDA‐International Diagnoses Classification (NANDA‐I), the Nursing Outcomes Classification (NOC) and the Nursing Interventions Classification (NIC) (Odenbreit, Müller‐Staub, Brokel, Avant, & Keenan, 2013) (for abbreviations, see Table 1). These three classifications are linked and are referred to as the NANDA‐I, NIC and NOC (NNN) taxonomies (Johnson et al., 2011). The NNN taxonomies are recognized by the ANA (2015b) for their use in Electronic Health Records (as interface terminologies), as multidisciplinary reference terminologies in the Systematized Nomenclature of Medicine—Clinical Terms (SNOMED CT) and as well as Logical Observation Identifier Names and Codes (LOINC) for communicating similar meaning across different software systems and different settings. The application of the NNN taxonomy relies on clinical decision‐making including diagnostic, therapeutic and ethical judgements (Gordon, ; Müller‐Staub et al., 2015). After implementation of the Advanced Nursing Process, nurses stated more accurate nursing diagnoses and performed more effective interventions that led to better patient outcomes (Müller‐Staub, Needham, et al., 2008; Pérez Rivas et al., 2016). However, previous studies demonstrated deficits in the application of the Advanced Nursing Process, including severe documentation errors. Missing assessments, inaccurate nursing diagnoses and inconsistencies between diagnoses, interventions and outcomes have been reported (Gershater et al., 2010; Halvorsen, Eide, Sortland, & Almendingen, 2016; Kebede, Endris, & Zegeye, 2017; Paans, Sermeus, Nieweg, Krijnen, & Schans, 2012). For instance, only 36% of nursing diagnoses recognized by Registered Nurses (RNs) were documented in the nursing records (Kobleder, 2011). A considerable consequence of such deficiencies is impaired clinical decision‐making, which leads to less positive nursing‐sensitive outcomes (Paans, Sermeus, Nieweg, & Schans, 2010; Saranto & Kinnunen, 2009; Zegers et al., 2011). Gershater et al. (2010) also reported negative medical, professional and economic consequences. Many factors can lead to rudimentary applications of the Advanced Nursing Process*.* These factors were specifically identified in individuals (deficient nurse knowledge and attitude), in the social context (organizational culture and institutional structures) and in financial limitations (Grol & Wensing, 2013). Nurses need patient‐related knowledge in addition to knowledge regarding diagnostic concepts, evidence‐based interventions and outcomes (Lunney, 2010; Paans, Nieweg, van der Schans, & Sermeus, 2011). Nurses’ attitude towards the nursing process is seen as a major influencing factor on its application (Ajzen, 2012; Romero‐Sánchez, Paloma‐Castro, et al., 2013; Romero‐Sánchez, Paramio‐Cuevas, et al., 2013) and on diagnostic prevalence and accuracy (Collins, 2013; Paans et al., 2011). In addition, electronic and handwritten nursing records have been identified as obstacles: nursing diagnoses have been inaccurately documented, and nurses have been found to be unfamiliar with SNLs (Conrad, Hanson, Hasenau, & Stocker‐Schneider, 2012; Paans et al., 2012). With respect to organizational factors, the attitude of supervisors towards nursing diagnoses has been reported in previous studies as a significant influencing factor (Axelsson, Björvell, Mattiasson, & Randers, 2006; Westendorf, 2007) and also, organizational factors, such as staff turnover (particularly of nurse leaders), nurse–patient ratio, workload level and nursing organization systems, influence nurses’ diagnostic competencies and their performance of the nursing process (Paans et al., 2011).

**Table 1:** Meaning of abbreviations

APN	Advanced Practice Nurse
GCR	Guided clinical reasoning
NANDA‐I	NANDA‐International Diagnoses Classification
NIC	Nursing Interventions Classification
NNN	Linked classifications of NANDA‐I, NIC and NOC
NOC	Nursing Outcomes Classification
PND	Positions on Nursing Diagnoses Scale
Q‐DIO (R)	Instrument Quality of Nursing Diagnoses, Interventions and Outcomes (revised)
SNL	Standardized nursing language

Several educational interventions were performed to develop nurses’ knowledge, their attitude and skills to improve the application of the Advanced Nursing Process. Nursing process‐based simulation trainings (Kim & Shin, 2016), with or without simulated patients or nursing records, were implemented (Bolstad, Xu, Shen, Covelli, & Torpey, 2012; Karadag, Caliskan, & Iseri, 2016; Lambie, Schwend, & Scholl, 2015) as well as multi‐day trainings with case studies/case discussions (Bruylands, Paans, Hediger, & Müller‐Staub, 2013; Müller‐Staub, Needham, et al., 2008), from 12 hr (Collins, 2013), over 5 days (Odutayo, Olaogun, Oluwatosin, & Ogunfowokan, 2013) to 10 days (Patiraki, Katsaragakis, Dreliozi, & Prezerakos, 2017). Each educational intervention has shown at least partial improvements in nurses’ knowledge, attitude and/or skills required for clinical reasoning and writing meaningful care plans.

### 2.1 Theoretical framework of the study

#### 2.1.1 Guided clinical reasoning

**Figure 1:**
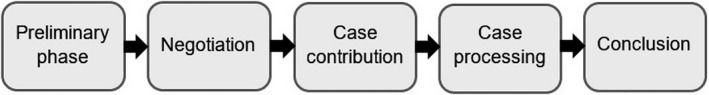
The five stages of case‐meeting GCR (Müller‐Staub & Stuker‐Studer, 2006)

Guided clinical reasoning will be used as study intervention. GCR is an educational approach aiming to improve nurses’ diagnostic competencies to state accurate nursing diagnoses and to link these with effective nursing interventions to achieve favourable patient outcomes. It is an interactive teaching method that is based on constructivist theories (Müller‐Staub, 2007; Siebert, 1999) and Balint's case supervision (Müller‐Staub, 1992). These approaches were further developed and combined with the diagnostic process by Müller‐Staub (Bruylands et al., 2013; Müller‐Staub, Needham, et al., 2008). GCR is applied in case meetings and contains five working stages (Figure 1). GCR must be led by a moderator with strict, straightforward rules. In the preliminary phase (a), several patients (termed cases) are briefly introduced to prepare all participants for the case meeting. In the second phase (b), a negotiation process leads to case selection. Then (c), the case provider further presents his/her case by freely telling those present about his/her experiences with the patient in an unstructured manner. In the case‐processing phase (d), the participants are individually asked by the moderator to communicate their spontaneous thoughts and associations. The moderator asks questions to aid his/her analysis, leading to hypotheses concerning the patients’ nursing diagnoses. In each round, the case presenter gives deeper insights on the patient by building on the participants’ reflections/assumptions. No discussion or problem‐solving suggestions are allowed at this stage. This “working phase” continues until a clear picture of the case arises. Finally, a meta‐perspective (e) and hypothetical nursing diagnoses are stated. In the case evaluation phase, the participants compare the hypothetical nursing diagnoses using SNL. The moderator asks questions such as, “Does the definition of the nursing diagnosis describe the case? Which of the related factors are seen in the presented case?”. After validating the hypothetical nursing diagnosis in comparison with the SNL, the participants are asked to choose appropriate, SNL‐based nursing outcomes and effective nursing interventions (Bulechek, Butcher, Dochterman, & Wagner, 2016; Doenges, Moorhouse, & Murr, 2014; Moorhead, Johnson, Maas, & Swanson, 2013). The results of the case meeting are put into practice by writing an SNL‐based care plan and evaluating it with the patient. By doing so, the Advanced Nursing Process is performed.

Only two studies (record audits) report GCR effects: (a) nursing assessments were improved, (b) nursing diagnoses were more specific and accurate, and (c) nursing interventions were more diagnosis/aetiology‐specific and therefore more effective (Bruylands et al., 2013; Müller‐Staub, Needham, et al., 2008). However, studies examining nurses’ knowledge, attitude and clinical performance before and after GCR are lacking. No clinical observational studies on the effects of GCR on performing the Advanced Nursing Process in practice are available, and patients’ perspectives have not yet been included.

#### 2.1.2 Evaluation model

**Figure 2:**
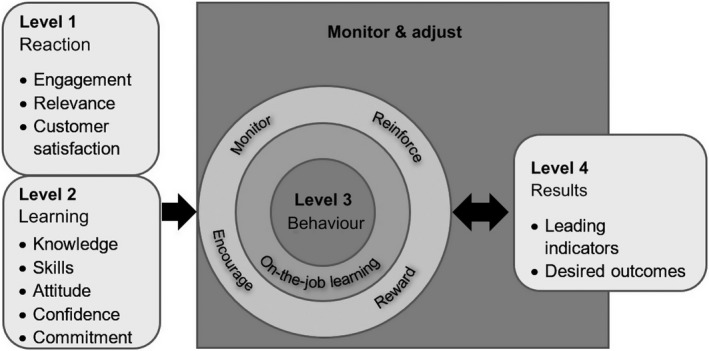
The New world Kirkpatrick model (Kirkpatrick Partners, 2010–2012)

The New world Kirkpatrick model (Figure 2) provides the framework to evaluate the effects of GCR. This model has been widely applied and published to evaluate education programmes; it contains four levels to develop a chain of evidence: Reaction (level 1), Learning (level 2), Behaviour (level 3) and Results (level 4) (Kirkpatrick & Kirkpatrick, 2008, 2010).

At level 1, a conventional satisfaction survey is conducted with the participants (e.g. nurses). The assumption is that participants can profit only if they are satisfied with the training. An additional satisfaction survey filled out by the supervisor of trainees (e.g. nursing director) is suggested to enhance the validity of results (Kirkpatrick & Kirkpatrick, 2010). At level 2, the knowledge, skills and attitude gained by the participants are examined. Level 1 and level 2 are measured in the learning field (e.g. training sessions). The level 3 evaluation addresses the effects of the GCR intervention on nurses’ behavioural changes in their field of work (their clinical performance on the hospital wards). The study participants, their supervisors and/or others that are familiar with nurses’ performance (e.g. patients, Advanced practice nurses [APNs]) are assessing the transfer results of the study intervention (Kirkpatrick, 1979). The effectiveness of GCR for the institution is measured at level 4: the desired outcomes (the quality of the Advanced Nursing Process). For levels 2–4, the model recommends quantitative pre–post‐test designs and the evaluation across all four levels is indispensable to build a chain of evidence. This means that the findings of data and method triangulation must be synthesized and compared. The evaluation of a programme's effectiveness based on Behaviour (level 3) or on Results (level 4) is useless without the inclusion of Learning (level 2) (Kirkpatrick & Kirkpatrick, 2008).

How are these levels addressed in this planned study? Satisfaction with the GCR intervention (Reaction—level 1) will be measured by a participant satisfaction survey, and the leadership perspective will be covered by an interview with the nursing director. A knowledge test and a self‐assessment instrument will be used to examine nurses’ knowledge and attitude towards the Advanced Nursing Process (Learning—level 2). APNs will observe nurses’ performance of the Advanced Nursing Process in practice to evaluate nurses’ clinical behaviour after GCR (Behaviour—level 3); additionally, patients will be interviewed on their experiences with the nurses’ knowledge, skills and attitudes. The results of the GCR intervention (level 4) will be evaluated by record audits with the Q‐DIO R instrument, which measures the accuracy of nursing diagnoses, the effectiveness of nursing interventions and the quality of patient outcomes. Furthermore, organizational factors will be collected to control possible confounders.

### 2.2 Objective

The aim of the planned study was to evaluate the effect of GCR on the quality of the *Advanced Nursing Process* by using Kirkpatrick's 4‐level evaluation model (Kirkpatrick & Kirkpatrick, 2008, 2010).

### 2.3 Research questions

The main research question is What effect does GCR have on the Advanced Nursing Process? According to the operationalized levels of the Kirkpatrick model, the detailed research questions are

- How do the participating nurses and the nursing director rate GCR? (Reaction)
- What effect does GCR have on nurses’ knowledge and on their attitude towards the Advanced Nursing Process in the learning field? (Learning)
- What effect does GCR have on nurses’ clinical performance and attitude towards the Advanced Nursing Process on the wards? How do patients describe their experience of the Advanced Nursing Process (nurses’ knowledge, skills and attitudes)? (Behaviour)
- What effect does CGR have on the accuracy of nursing diagnoses (including frequency and variety), the effectiveness of nursing interventions and the quality of nursing outcomes? (Results)
- Are the findings of data and method triangulation (satisfaction, knowledge, attitude, Q–DIO R scores, APNs and patients’ perspective on nurses’ clinical performance) mutually supportive to build a chain of evidence?

### 2.4 Hypotheses

- After GCR, nurses in the experimental group will have a significantly higher total score in the knowledge test and a significant more positive attitude towards the Advanced Nursing Process on the Positions on the Nursing Diagnoses (PND) Scale than those in the control group.
- After GCR, in the experimental group the congruence between observations of direct care, patients’ statements and nursing records will be greater than that in the control group.
- After GCR, in the experimental group the quality of nursing diagnoses, interventions and outcomes in record audits (measured by Q‐DIO R) will be significantly higher than that in the control group.

## 3 METHODS

### 3.1 Study design and setting

A complex experimental intervention study using data and method triangulation will be performed. A complex intervention contains a “number of interacting components […], number and difficulty of behaviours required by those delivering or receiving the intervention, number of groups or organizational levels targeted by the intervention, number and variability of outcomes [and] degree of flexibility or tailoring of the intervention permitted” (Craig et al., 2008). General teaching and learning processes as well as GCR are guided and influenced by teachers’ and participants’ behaviour and various context conditions. Furthermore, the knowledge transfer from the learning field to the work field including organizational influencing factors will be considered, and therefore, several outcomes on different levels will be measured. Quantitative methods will be used to take knowledge tests and self‐assessments on nurses’ attitude towards the Advanced Nursing Process. The quality of the Advanced Nursing Process—including the accuracy, frequency and variety of nursing diagnoses—will be evaluated by nursing record audits with the Q‐DIO R instrument. However, a general criticism is how the nursing reality is represented in the nursing records. Therefore, to the objectives of level 3 evaluation, observations of nurses’ clinical performance of the Advanced Nursing Process, interviews with patients and qualitative document analyses will be performed to verify the Q‐DIO results (Kirkpatrick & Kirkpatrick, 2008, 2010).

The study will be conducted in a Swiss public hospital. From a total of 12 wards, seven fulfil the inclusion criteria in that they are acute geriatric (2), internal medicine (3) or surgical (2) inpatient wards. By sealed‐envelope drawings, three wards will be randomly allocated to the experimental group, three to the control group and one will be excluded.

### 3.2 Sample and eligibility criteria

RNs, the nursing director, nursing records and patients will be included in this intervention study.

#### 3.2.1 Registered nurses

All RNs of the six included wards will be recruited (*N = *92) for attitude measurements. Using stratified convenience sampling, a third (*N = *34) of the RNs will participate as experimental group in GCR training (*N = *17) or as control group (*N = *17). Both groups should contain a similar grade‐skill mix of ward managers, nurse instructors, Advanced Nursing Process mentors and regular RNs. All ward managers will be included. The aim is to empower them for taking responsibility on the Advanced Nursing Process. As role models, their knowledge, attitude and behaviour should spread to their teams. The inclusion criteria are being in a leadership, teaching or clinical position, applying the Advanced Nursing Process and understanding the main national language.

#### 3.2.2 Nursing records

By using a web‐based random generator, a sample of nursing records (*N = *180) will be drawn (https://www.random.org/). Nursing records covering at least 4 days and at least one nursing diagnosis will be selected from all six wards (experimental group *N = *90, control group *N = *90). The sample‐size calculation was performed using *t*‐tests for unpaired samples (one‐sided) and bases on previous results for using the Q‐DIO for all three sub‐concepts of the instrument (nursing diagnoses, interventions and outcomes) (Müller‐Staub, Needham, et al., 2008). An effect size of 0.5 (mean) on a scale from 0–4 is assumed. For nursing diagnoses, 32 nursing records per group are required; we assumed an effect size of 0.63 (power 80%). For nursing interventions, 45 records (effect size, 0.53; power, 80%) are required and for nursing outcomes, 44 records (effect size, 0.47; power, 70%) are required. To ensure an even distribution in the experimental and control groups, 15 nursing records will be selected per ward (*N = *45 records before and 45 records after the study intervention, totalling *N = *90) for each group (Bortz & Schuster, 2016).

#### 3.2.3 Patients

From the randomly selected nursing records, four patients per ward will be included (*N = *24) to participate in interviews and clinical observations. The inclusion criteria are patients older than 18 years, physically and mentally capable to take part in the interview (e.g. without serious symptoms and completely oriented) and being able to answer interview questions in the main national language.

### 3.3 Study intervention

Guided clinical reasoning will be applied to the experimental group on GCR training days. The intervention comprises four single days at a duration of 5 months, and each GCR day comprises 7 hr. The participating nurses will receive materials on the Advanced Nursing Process including presentations, information about relevant websites, assigned reading and work assignments (Table 2). These documents will be distributed in printed form or as PDF files. Prior to starting GCR, the participating nurses will be asked about their previous knowledge, experiences, learning needs and interests. On each of the four GCR training days, several learning methods will be applied such as a GCR case‐meeting, presentations, small‐group work and discussion rounds. Additional work assignments (transfer tasks) to put the newly gained knowledge into clinical practice include formulating and documenting SNL‐based, accurate nursing diagnoses for a patient in the PES format (problem definition, aetiological factors and signs/symptoms) and writing coherent care plans. Verifying nursing diagnoses and the care plan with the patient is strongly recommended.

**Table 2:** Standardized educational intervention protocol GCR

Date	Objectives/contents	Assigned reading
Day 1	Advanced Nursing Process and nursing diagnoses (objectives, purpose, advantages and disadvantages, terms/definitions/concepts, references to nursing science, historical background, prospects, frequent nursing diagnoses, components of nursing diagnoses—PES format) Diagnostic process (steps, exercises, difficulties) NANDA‐I Taxonomy II (application of the work by Doenges et al. (2014)	History of the nursing process Clinical decision‐making in the diagnostic process by means of case‐meeting GCR Nursing process for leaders
Day 2	Reflections on assigned readings and examination of work assignments Exploration/definition of concepts (e.g. Advanced Nursing Process and nursing diagnostics) Problem areas in practical implementation Repetition of nursing diagnostic steps and clinical decision‐making Historical background and prospects NANDA‐I Taxonomy II (application of the work by Doenges et al. (2014)	Quality improvement by nursing diagnoses? Systematic review: nursing diagnoses, interventions and outcomes—application and impact on nursing practice Evaluation study relating to the implementation of nursing diagnoses, interventions and outcomes
Day 3	Reflections on assigned readings and examination of work assignments Reflecting economization and practice Diagnostic process: components of critical thinking, clinical decision‐making and difficulties Stating, validating and prioritizing nursing diagnoses	NNN‐nursing assessment Implementation project regarding electronic assessment Repetition of the first handout is recommended for evaluation Optional articles on critical thinking, GCR and electronic documentation
Day 4	Reflections on assigned readings and the work assignments Exercises relating to nursing diagnostic steps and application of the work by Doenges et al. (2014) Stating, validating and prioritizing nursing diagnoses Intervention study of nursing diagnoses, interventions and outcomes Repetition and conclusions	

In addition to the GCR training days, all nurses in the experimental group will participate in short GCR case meetings (0.5 hr) on their wards. All available nurses on duty (on average, eight persons) participate in these meetings, where one nurse acts as the case presenter. While GCR trainings will take place in a seminar room of the hospital, short GCR case meetings will be conducted in the field of work, that is, on the wards. GCR case meetings will be conducted three to five times during the nurses’ dayshift on the experimental wards. On the control wards, the nurses will not participate in a training and the current introduced nursing process including NANDA‐I‐nursing diagnoses will be continued. The same number of regular case meetings will be performed for the same duration but without GCR.

An internationally experienced expert (RN, nurse pedagogue and nursing scientist) will perform the GCR intervention.

### 3.4 Expected outcomes

The primary outcomes are the level of the nurses’ knowledge, the level of their attitude, satisfaction with GCR, the quality of nursing diagnoses, interventions and outcomes and the frequency and variety of NANDA‐I‐nursing diagnoses (Doenges et al., 2014; NANDA‐I, 2016). The secondary outcomes are nurses’ behaviour in the field of work (clinical performance of the Advanced Nursing Process evaluated by observations and by patient interviews) and mutual comparisons of all findings.

### 3.5 Data collection

#### 3.5.1 Instruments

##### 3.5.1.1 Knowledge test

A knowledge test will be distributed to the experimental and control groups on the first and last GCR days. This test was previously developed for evaluating similar GCR training sessions. After minor adjustments and a pre‐test, it contains eleven items: six qualitative knowledge questions, two supplementary control questions on content not covered by the GCR sessions and three self‐evaluations on the learning success. For the knowledge items, the maximum attainable test score is 58 points, and for the control items, the maximum score is thirteen points. Participants take the test silently by hand under expert supervision. A maximum of 30 min is allowed for its completion and the participants fill in a personal four‐digit number (e.g. the last digits of their mobile phone) to code the test(s), thus allowing anonymous, matched comparisons before and after the GCR training.

##### 3.5.1.2 Positions on the Nursing Diagnoses Scale (PND)

The nurses’ attitude towards nursing diagnoses will be measured using the self‐assessment instrument PND (Lunney & Krenz, 1994), which contains 20 items with positive and negative pairs of bipolar attitude adjectives on a 7‐point Likert scale. The total score ranges from 20–140; the more positive the attitude, the higher the total score. The German PND version has been previously evaluated (Leoni‐Scheiber, Gothe, & Müller‐Staub, 2016). The psychometric properties of the original PND showed favourable results: its content validity exceeded an agreement of 90% by four expert raters (Lunney & Krenz, 1994), the test–retest reliability as measured by intraclass correlation coefficient was 0.90 (95% CI [0.87, 0.92]), and the internal consistency as measured by Cronbach's alpha coefficient was 0.96 (Romero‐Sánchez, Paloma‐Castro, et al., 2013). The construct validity was measured by confirmatory factor analysis and showed highly correlated factors in the three‐factor model solution (≥0.96) (D'Agostino et al., 2016). The PND requires 5 min to complete and will be filled out by the participating nurses in handwriting on the first and last GCR days and 3 months later on their respective wards. To assure anonymity and to ensure that the results can be compared with the knowledge tests of each nurse, the participants will be asked to fill in the same four‐digit number as in the other instrument.

##### 3.5.1.3 Satisfaction survey

This survey will be applied to assess the participants’ satisfaction with GCR. It contains seven items with three‐ and four‐point scale answer options (e.g. very good, good and bad) and a bipolar eight‐point Likert scale with three pairs of opposite views (e.g. GCR content has practical relevance or is too theoretical). Additional free space allows adding comments to each item. This survey will be completed on the last GCR day by hand and requires 5–7 min for completion. The participants use the same personal four‐digit number as for the other instruments. To obtain insight into satisfaction with GCR from a leadership perspective, a semi‐structured interview with the nursing director will be conducted and recorded (Kirkpatrick & Kirkpatrick, 2010).

##### 3.5.1.4 Clinical observation guide

The semi‐structured guide for nonparticipatory observations by four APNs covers four areas: patients’ central nursing problems that the nurses address, performed nursing interventions and the effectiveness of interventions in affecting nursing problems/diagnoses and nursing outcomes. The observations will be recorded by hand, and the observation duration ranges from 30–60 min and occurs on the same observation day.

##### 3.5.1.5 Patient interview guide

The guide for the semi‐structured patient interviews starts with an introductory question on their overall satisfaction with nursing care. Further questions address patients’ main nursing care needs, their experiences of nurses’ interest in and skills regarding their needs, received nursing interventions, the patients’ main aims/expected outcomes and their inclusion into nursing care planning. Patients will address the care needs that they are able to recognize. The interview notes will be recorded by hand by the interviewing APN, and all participating APNs have been previously trained in data collection and in the use of the guide(s).

##### 3.5.1.6 Quality of diagnoses, interventions and outcomes (Q‐DIO)

The Q‐DIO instrument is revised for this study to assess the quality of the Advanced Nursing Process using record audits (Müller‐Staub, Lunney, et al., 2008; Müller‐Staub et al., 2009, 2010). The Q‐DIO contains a three‐point scale (0–2) for the 12 nursing assessment items and a five‐point scale (0–4) for the other three sub‐concepts (nursing diagnoses, interventions and outcomes). The higher the point total, the higher the quality of the Advanced Nursing Process. The original Q‐DIO has been validated in several studies and shows favourable validity and reliability; the internal consistency as measured by Cronbach's alpha for each sub‐concept ranged from 0.83–0.99, the test–retest reliability as measured by kappa was 0.95, and the inter‐rater reliability as measured by Fleiss’ kappa was 0.94 (Linch, Müller‐Staub, Moraes, Azzolin, & Rabelo, 2012; Linch et al., 2015; Müller‐Staub, Lunney, et al., 2008; Müller‐Staub et al., 2009, 2010). All nursing records will be evaluated by each Q‐DIO item and recorded in an SPSS data mask (SPSS Inc.). To ensure a consistent approach of its application, memos will be written, and the instrument developer is consulted.

##### 3.5.1.7 Tool for demographic and organizational data

To control for influencing factors, all participating nurses will be asked about their gender, highest education grade, years of practical experience and previous seminars on the nursing process. To control for system influences, the number of beds, capacity, patients’ length of stay, nurse–patient ratio, grade and skill mix, staff turnover and organizational system characteristics will be collected from each ward by the principal investigator using standardized data collection forms.

#### 3.5.2 Measurement time points before and after the GCR study intervention

**Figure 3:**
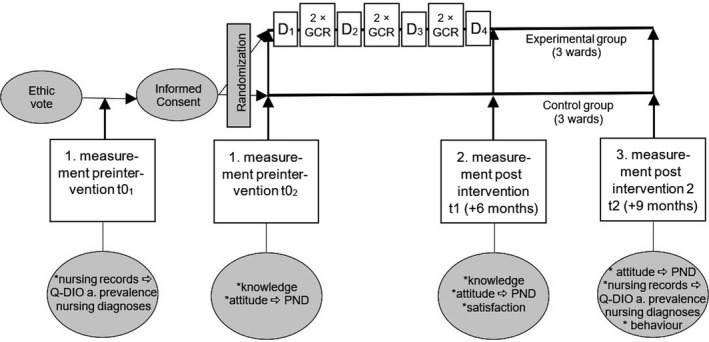
Flow chart of the study schedule. D_1 _‐ D_4_ = Days 1–4 of guided clinical reasoning training days; GCR = GCR case meetings on the wards; *t*0_1_ + *t*0_2_ = baseline

Data collection will occur at three time points; the flow chart in Figure 3 presents all data collection methods and time points. As baseline measurement before the GCR intervention (*t*0_1_ + *t*0_2, _illustrated in boxes): a sample of nursing records will be drawn, and nurses of the intervention and control group perform the knowledge tests and all nurses of the participating wards fill out the PND scales (illustrated in circles). After GCR, the participants complete knowledge tests and PNDs for the second time and complete the satisfaction survey (*t*1). After ending of the GCR intervention, all nurses complete the PND a second and a third time. At that time, a second sample of nursing records will be drawn. In addition, patient care will be observed (nurses’ clinical performance) and patients are interviewed to cross‐validate the Q‐DIO R results on the quality of the Advanced Nursing Process as described in the nursing records.

### 3.6 Data analyses

The knowledge test and nurses’ attitude (PND scale) will be analysed using descriptive and inductive statistics. Single‐item and total scores will be compared between the two measurement points (Wilcoxon signed‐rank test) and between the experimental and control groups (Mann–Whitney *U* tests). The scores of supplementary control items for the knowledge test will be separately analysed and compared between the two measurement points and the two groups. The results of the three nurses’ attitude measurements (*t*0_2_, *t*1 and *t*2) will be evaluated using Friedman tests (Bortz & Schuster, 2016; Clauß, Finze, & Partzsch, 2011). The free‐text item answers in the knowledge test will be analysed using Mayring's (2010) seven‐step content analyses.

Participant satisfaction will be descriptively analysed based on absolute and relative frequencies and distributions. Content analyses (Mayring, 2010) will be applied to the interview with the nursing director and to the free‐text data from the satisfaction surveys.

The Q‐DIO R scores will be summed for each sub‐concept. If the data are normally distributed, unpaired *t*‐tests are applied to compare pre‐ and postintervention scores and to compare the experimental and control groups. The frequencies and patterns (variety) of the NANDA‐I nursing diagnoses will be compared between the two measurement time points and between the experimental and control groups.

Correlations among nurses’ knowledge, attitude, Q‐DIO R scores, demographic and organizational characteristics will be analysed depending on level of data and testing requirements by Pearson product–moment correlations, Spearman correlations or chi‐squared tests. All tests will be evaluated one‐sided (levels of significance 0.05) using IBM SPSS Statistics 24 (SPSS Inc.).

The qualitative evaluation of nurses’ clinical behaviour will be performed by within‐ and cross‐case analyses (Kuckartz, 2016). Qualitative observations, interviews and nursing records will be analysed using the first and second coding cycles (Saldaña, 2015). Decoding (open) is applied in the first cycle, and encoding (deductive) is applied in the second cycle. For the qualitative data analyses, MAXQDA 12 software (VERBI GmbH, 2015) will be used.

Data and method triangulation will take place by applying Kirkpatrick's four levels. First, the findings of participants’ satisfaction survey and directors’ interview will be compared to verify whether these sources are mutually supportive (Reaction). Second, a data synthesis of findings from observations, patient interviews and nursing records (within‐ and cross‐case analyses) will be performed to evaluate nurses’ clinical performance of the Advanced Nursing Process (Behaviour). Finally, the chain of evidence (according to Kirkpatrick) will be verified as follows: a positive lower level is prerequisite for the next higher level; for example, a positive knowledge test (Learning) is a prerequisite for positive clinical performance (Behaviour).

### 3.7 Ethical considerations

This study was authorized by the regional ethics committee in April 2016 (Nr. PB_2016_00990), and data collection has started. The study will be executed in conformity with the Declaration of Helsinki (WMA, 2013) and with Good clinical practices (Altpeter et al., 2005).

The nurses and the nursing director will be asked for voluntary participation. Nursing records from the archives (pre‐intervention data) were permitted to be retrieved without patients’ informed consent. Written informed consent will be obtained for postintervention document analyses, for patient observations and for interviews. Comprehensive written and oral information on the study aim and procedures, together with descriptions of the patients’ rights as study participants, will be provided at least 1 day before data collection.

### 3.8 Validity and reliability/rigour

This study protocol adheres to the CONSORT statement (Schulz, Altman, & Moher, 2010), and the detailed description of the intervention is provided according to the TIDieR checklist (Hoffmann et al., 2014). The applied measurement instruments (PND and Q‐DIO R) have been psychometrically tested and showed good results (D'Agostino et al., 2016; Leoni‐Scheiber et al., 2016; Linch et al., 2015; Lunney & Krenz, 1994; Müller‐Staub et al., 2009, 2010; Romero‐Sánchez, Paloma‐Castro, et al., 2013). The satisfaction survey and the knowledge test were piloted by experts and modified before application. One expert will perform the study intervention to ensure it is strictly performed as described.

The data collection training for the four APNs is standardized, and these participating researchers are trained together to ensure that they receive the same information on using the observation and interview guides. With respect to social desirability, the patients will be assured that their care will not be affected by observations nor by their interview responses. Therefore, the APNs will not perform observations and interviews in their own clinics and all data will be anonymized and coded.

## 4 DISCUSSION

This protocol outlines a proposed experimental intervention study using data and method triangulation to measure the effects of GCR. First, the Advanced Nursing Process using SNLs and GCR are described as key concepts. Second, the New world Kirkpatrick evaluation model and how it will be applied is presented. This model's predecessor was often reduced to supporting management in terms of the return on investment. Using the model in mechanistic ways would be incompatible with its real aims and for complex interventions such as GCR (Seeber, Krekel, & Buer, 2000). Evaluating all levels by methodological and data triangulation allows using of a variety of data sources (e.g. nurses, records, patients and organizational data). This approach provides broad insights from different perspectives (Creswell, 2014) into the quality of the Advanced Nursing Process, and combining quantitative and qualitative methods can strengthen the evaluation of intervention effects (Tashakkori & Teddlie, 2010). For these reasons, the authors believe that Kirkpatrick's 4‐level model is particularly well suited for the proposed study. It is anticipated that it will enable the development of a comprehensible chain of evidence supporting GCR effects on the Advanced Nursing Process*.*

### 4.1 Limitations

The generalizability of the expected results will be restricted due to the sample sizes in this planned study. Despite the recommendations of Kirkpatrick and Kirkpatrick (2008) to also analyse nurse behaviour before and after study interventions, only postintervention observations are possible due to the limited available resources. All other aspects will be measured before and after GCR. By consistently following the rules for intervention studies (manipulation, randomization and control), robust results can be anticipated.

## 5 CONCLUSION

This study protocol has outlined the rationale and design to examine the effectiveness of GCR. If the training participants and the nursing director are satisfied with GCR and if it leads to enhanced nurse knowledge, attitudes and clinical performance as well as to a higher quality of nursing diagnoses, interventions and outcomes, then replications in other settings and places can be suggested. If the study hypotheses are supported, recommendations can be provided on using GCR in practice to enhance the application of the Advanced Nursing Process.

## CONFLICT OF INTEREST

No conflict of interest has been declared by the authors.

## AUTHOR CONTRIBUTIONS

CLS, HM and MMS made substantial contributions to conception and design, or acquisition of data, or analysis and interpretation of data; involved in drafting the manuscript or revising it critically for important intellectual content; given final approval of the version to be published. Each author should have participated sufficiently in the work to take public responsibility for appropriate portions of the content; and agreed to be accountable for all aspects of the work in ensuring that questions related to the accuracy or integrity of any part of the work are appropriately investigated and resolved.
